# Arsenic exposure is associated with pediatric pneumonia in rural Bangladesh: a case control study

**DOI:** 10.1186/s12940-015-0069-9

**Published:** 2015-10-23

**Authors:** Christine Marie George, W. Abdullah Brooks, Joseph H Graziano, Bareng A. S. Nonyane, Lokman Hossain, Doli Goswami, Khalequzzaman Zaman, Mohammad Yunus, Al Fazal Khan, Yasmin Jahan, Dilruba Ahmed, Vesna Slavkovich, Melissa Higdon, Maria Deloria-Knoll, Katherine L. O’ Brien

**Affiliations:** 1 Department of International Health, Johns Hopkins Bloomberg School of Public Health, Baltimore, MD USA; 2 International Centre for Diarrhoeal Disease Research, Bangladesh (icddr,b), Dhaka, Bangladesh; 3 Department of Environmental Health, Mailman School of Public Health, Columbia University, New York, NY USA; 4 Department of International Health, Program in Global Disease Epidemiology and Control, Johns Hopkins Bloomberg School of Public Health, 615 N. Wolfe Street, Room E5535, Baltimore, MD 21205-2103 USA

**Keywords:** Arsenic, Pneumonia, Pediatric population, Bangladesh

## Abstract

**Background:**

Pneumonia is the leading cause of death for children under 5 years of age globally, making research on modifiable risk factors for childhood pneumonia important for reducing this disease burden. Millions of children globally are exposed to elevated levels of arsenic in drinking water. However, there is limited data on the association between arsenic exposure and respiratory infections, particularly among pediatric populations.

**Methods:**

This case control study of 153 pneumonia cases and 296 controls 28 days to 59 months of age in rural Bangladesh is the first to assess whether arsenic exposure is a risk factor for pneumonia in a pediatric population. Cases had physician diagnosed World Health Organization defined severe or very severe pneumonia. Urine collected during hospitalization (hospital admission time point) and 30 days later (convalescent time point) from cases and a single specimen from community controls was tested for urinary arsenic by graphite furnace atomic absorption.

**Results:**

The odds for pneumonia was nearly double for children with urinary arsenic concentrations higher than the first quartile (≥6 μg/L) at the hospital admission time point (Odd Ratio (OR):1.88 (95 % Confidence Interval (CI): 1.01, 3.53)), after adjustment for urinary creatinine, weight for height, breastfeeding, paternal education, age, and number of people in the household. This was consistent with findings at the convalescent time point where the adjusted OR for children with urinary arsenic concentrations greater than the first quartile (≥6 μg/L) was 2.32 (95 % CI: 1.33, 4.02).

**Conclusion:**

We observed a nearly two times higher odds of pneumonia for children with creatinine adjusted urinary arsenic concentrations greater than the first quartile (≥6 μg/L) at the hospital admission time point. This novel finding suggests that low to moderate arsenic exposure may be a risk factor for pneumonia in children under 5 years of age.

**Electronic supplementary material:**

The online version of this article (doi:10.1186/s12940-015-0069-9) contains supplementary material, which is available to authorized users.

## Introduction

Millions of people in countries around the world are exposed to elevated levels of arsenic in drinking water. In Bangladesh alone, it has been estimated that more than 35 million people are exposed to naturally occurring arsenic in drinking water at levels exceeding the World Health Organization guideline of 10 μg/L [1]. This arsenic is believed to result from arsenic-rich iron oxides in sediments being dissolved and released into the groundwater aquifer [2, 3]. There is a growing body of literature linking arsenic exposure to adverse respiratory outcomes including reduced lung function [4–6], cough [7–13], and less conclusively lower respiratory tract infections [14], bronchitis [12, 15, 16], and pulmonary tuberculosis mortality [17]. However, most studies have been conducted in adult populations, and there have been no studies to date evaluating the association between arsenic and pneumonia in pediatric populations. Pneumonia is the leading cause of death for children under 5 years of age globally, making research on modifiable risk factors for childhood pneumonia important for reducing this disease burden [18].

Although there are no published studies assessing the association between arsenic and pneumonia there are four cohort studies that have investigated the association between arsenic exposure and pediatric respiratory diseases [14, 19–21]. A cohort study in Matlab, Bangladesh found that maternal urinary arsenic concentrations during gestation were negatively associated with interleukin-7, lactoferrin, and child thymic index at 12 months, and positively associated with the number of days of acute respiratory infections at 6–12 months [14]. A second cohort study in Matlab found that infants of mothers with urinary arsenic concentrations in the highest quintile during gestation had a significantly increased relative risk of lower respiratory tract infection at 12 months compared to those in the lowest quintile [22]. In New Hampshire, a cohort study reported a significant association between *in utero* arsenic exposure and upper respiratory infections in 4 month old infants [21]. Finally, a fourth recently published cohort study from Matlab, Bangladesh found a significant association between *in utero* and early life arsenic exposure and wheezing and shortness of breath [20]. These cohort studies were important to the field because they demonstrated a significant association between *in utero* arsenic exposure and subsequent respiratory infections. However, one potential limitation was the use of caregiver reported respiratory symptoms which could be subject to reporting bias, particularly if caregivers knew the arsenic status of their household wells.

Animal studies have found that arsenic in drinking water causes immune suppression that is suspected to affect the pulmonary defense system [23–28]. In Kozul et al. arsenic exposure was associated with increased morbidity and pulmonary influenza virus titers in mice exposed to H1N1 virus [29]. In another animal study, tumor necrosis factor-α (TNF-α) and IL1b were significantly altered in the lungs of arsenic exposed mice [30]. Arsenic has also been shown to interfere with the Janus kinase-signal transducer and activator of transcription (JAK-STAT) pathway, essential for mediating cytokine receptor signaling pathways and the regulation of cell growth, by suppressing cytokine function and production [27]. In human studies, a study in Mexico found that increased urinary arsenic was significantly associated with reduced percentages of CD4 T cells and interleukin (IL)-2 secretion levels [31]. Consistent with this finding, a study in West Bengal found that arsenic exposure was associated with reduced T-cell proliferation and cytokine secretion (TNF-α,Interferon-γ, IL-2, IL-10) [32]. A study in Bangladesh found that arsenic exposure from drinking water during pregnancy was associated with lower breast milk concentrations of IL-7 and lactoferrin [14]. Furthermore, another recent study in Bangladesh found that prenatal arsenic exposure was associated with a decreased percentage of CD4 T cells in cord blood [33]. These findings suggest that arsenic exposure may alter the activation of CD4 T cells resulting in reduced cytokine secretion and immune suppression.

Given this evidence, we hypothesize that arsenic exposure suppresses immune function in children making them more susceptibility to pneumonia. In an initial attempt to test our hypothesis, we have conducted the first study to assess the relationship between arsenic exposure and pneumonia in a pediatric population.

## Patients and methods

This study was nested within the Pneumonia Etiology Research for Child Health (PERCH) study, and was undertaken at the Matlab, Bangladesh site. PERCH is a case–control study conducted in 7 countries (Bangladesh, Thailand, Kenya, The Gambia, Mali, Zambia, and South Africa) to determine the etiology of severe and very severe hospitalized pneumonia and associated risk factors in children under 5 years of age [34]. These sites were selected because they were regions predicted to have the most severe pneumonia in children in 2015. In Bangladesh there were two PERCH sites, one in urban Dhaka, Bangladesh and one in rural Matlab, Bangladesh. Data from the Dhaka site was not included in our current study because this site uses a low arsenic municipal water supply.

Matlab is a rural area in the Chandpur District of Bangladesh, and is part of the International Centre for Diarrhoeal Disease Research, Bangladesh (icddr,b) Health and Demographic Surveillance System (HDSS). The Matlab HDSS maintains a registry of births, deaths, migrations, and vital health outcomes for over 220,000 individuals [35]. Previous studies conducted in the Matlab HDSS report that 70 % of the 13,286 wells in the HDSS contain arsenic exceeding the World Health Organization (WHO) guideline of 10 μg/L [35]. All individuals living within the Matlab HDSS are registered in the HDSS database. New residents are registered when they enter the study area.

All severe and very severe pneumonia cases were recruited from Matlab icddr,b Hospital located within the HDSS catchment area. There are no other hospitals in the Matlab HDSS area. Cases were defined as children 28 days to 59 months of age hospitalized with physician diagnosed World Health Organization (WHO) defined severe or very severe pneumonia. Severe pneumonia was defined as children presenting with a cough or difficulty breathing, and lower chest wall in-drawing. Very severe pneumonia was defined as children with a cough or difficulty breathing and any of the following symptoms: central cyanosis, unable to feed, vomiting, convulsions, lethargy, impaired consciousness, or head nodding [36]. Case inclusion criteria were that they lived within the Matlab HDSS catchment area. The case exclusion criteria were if the child had been hospitalized in the past 14 days or if the child had been discharged from the Matlab icddr,b hospital in the past 30 days as a PERCH case. Cases were enrolled over a 21 month period including enrollment during nights and weekends to ensure that they were representative of the disease occurrence in the population.

Controls were frequency matched to cases based on the following age strata: 28 days to <6 months, 6 to <12 months, 12 to <24 months, and 24–59 months. Controls were randomly selected within these age strata from their homes using the Matlab HDSS database. If a child met the study eligibility criteria they were ask to come to Matlab icddr,b hospital for a clinical examination by a physician. The inclusion criteria for controls were that they were children 28 days to 59 months of age and living within the Matlab HDSS catchment area. The control exclusion criteria were the following: (1) child had been hospitalized in the past 14 days; (2) child had been discharged from the Matlab icddr,b hospital in the past 30 days as a PERCH case; (3) child appeared very sick requiring urgent medical attention; or (4) child was found to have WHO defined severe or very severe pneumonia during their clinical examination. A minimum of 12 community controls were recruited per month; and in months when more than 12 cases were enrolled, additional controls were enrolled so as to frequency-match by season to cases [34].

Elimination of ingested arsenic occurs predominately via the urine [37–39]. Urinary arsenic has been found to be a reliable biomarker of chronic arsenic exposure and is relatively stable over time if the source of drinking water remains constant [40–43]. Two urine samples were collected from cases: after confirmation of PERCH eligibility at icddr,b Matlab hospital (“hospital admission”) and approximately 30 days after the hospital admission date (“convalescent”). Controls had a single urine specimen collected; this took place during their clinical examination at the icddr,b Matlab hospital for the study enrollment activities. All urine samples were collected from study participants in 50 ml urine bags, transferred to two 5 ml acid washed tubes, and frozen at −20 °C at the local laboratory in Dhaka, Bangladesh until shipment on dry ice to the Trace Metals Core Laboratory at Columbia University for analysis. Total urinary arsenic concentration was measured by graphite furnace atomic absorption, using a Perkin-Elmer Analyst 600 graphite furnace system (Waltham, MA) with a detection limit of 2 μg/L [44]. Urinary creatinine was quantified using a method based on the Jaffe reaction [45]. This measure was used to adjust each child’s urinary arsenic concentration for their hydration status [46].

Our laboratory participates in the Interlaboratory Comparison Program for Metals in Biological Matrices run by The Centre de Toxicology du Quebec (CTQ). We receive samples of unknown arsenic concentration five times a year. Each batch of samples contains three urines with varying arsenic concentrations, which we analyze and report back to CTQ. The coefficient of correlation between reported and target values for 2013, when PERCH study urine samples were analyzed, was 0.99 with a z-score of 1.4 (<2 = satisfactory). Every day after initial calibration, three urine samples with known concentrations from CTQ are analyzed. They have to be within the established range for the instrument to continue running the unknown samples. Concentrations of known urine samples are chosen to cover the low, medium, and high range of the calibration curve. Throughout the day a medium quality control sample (QCS) is analyzed every 10 samples, and if it’s out of the established range, instrument recalibration is performed.

A questionnaire was administered to the child’s caregiver to obtain information on household socio-demographic characteristics and the child’s health status. Breastfeeding in the prior week was categorized as the following: exclusive, mixed with other foods or fluids, and none. Paternal education was categorized as no formal education, 1–5 years of education, 5–10 years of education, and greater than 10 years of education. Information was also collected on the number of people living within the household, this variable was log transformed in the logistic regression models. In addition, research assistants trained in standardized anthropometry, measured the child’s weight and height. Height and weight measurements were used to calculate z-scores according to the WHO child growth standards [47]. Wasting was defined as z-scores less than −2 SD for weight for height.

Our sample for this nested case control study within the PERCH study was based on the number of pneumonia cases recruited at the Matlab, Bangladesh site between January 2012 and September 2013. We estimated there would be approximately 178 cases (both severe and very severe pneumonia) and 226 controls enrolled into the PERCH study at the Matlab site over our study period. We conducted our power calculation in G*Power 3.1.3. Based on this anticipated sample size of 178 cases and 226 controls, we would have been able to detect a true difference in mean urinary arsenic concentrations in cases and controls of 17 µg/L creatinine, adjusted for creatinine concentrations, with a power of 80 % and a type 1 error of 0.05.

Logistic regression was used to determine if arsenic exposure, assessed by urinary arsenic concentrations, significantly increased the odds of being a pneumonia case. The main predictor, urinary arsenic concentration, was divided into quartiles. Covariates that had a *p*-value less than 0.1 or changed the ORs of pneumonia by more than 5 % in a model with the outcome and urinary arsenic were included in the multivariable logistic regression model as potential confounders. We compared both the hospital admission case urine and the convalescent case urine with the control urine. Data was divided into quartiles at the hospital admission time point using the hospital admission case urines and the control urines. Quartiles at the case convalescent time points were calculated using the case convalescent urine samples and the control urine samples. Because a small proportion of cases (<12 %) had very severe pneumonia, we included all cases, regardless of severity, in a single category in the main analysis. A sensitivity analysis was also performed to investigate the impact of very severe pneumonia on our observed associations. For this sensitivity analysis all regression models were performed without *very* severe pneumonia cases in the models. All analyses were performed using SAS, version 9.3 (SAS Institute Inc., Cary, NC, USA).

Informed consent from a guardian was obtained for cases and controls. All study procedures were approved by the research ethics committees of icddr,b and Johns Hopkins Bloomberg School of Public Health.

## Results

A total of 205 cases and 306 controls were recruited for the PERCH study at the Matlab, Bangladesh site between January 2012 and September 2013. Ninety-eight percent of cases (201/205) produced a urine sample at icddr,b Matlab hospital at the hospital admission timepoint, and 75 % (153/205) at the convalescent timepoint. Ninety-seven percent (296/306) of controls produced a urine sample. Cases and controls differed significantly by gender, weight for height, paternal education, and floor type of household (Table 1). Most children (95 %) lived in households where shallow wells were the main source of drinking water. Four case children did not produce a urine sample during our convalescent visit, 8 children migrated outside the study area or could not be located, two children died, three children did not produce enough urine for sample analysis, and 31 children had their convalescent visit after our study period had ended. There were no significant differences observed in demographic characteristics between cases which provided a urine sample at the convalescent time point, and those that did not (Additional file 1: Table S1). The mean duration of difficulty breathing for cases was 2 days (range 1–7 days). Thirty four percent of the 153 cases enrolled were fully vaccinated for Haemophilus Influenzae Type b (Hib) (>3 doses or ≥1 if the child is 12 months of age or older), and 66 % of the 296 controls were fully vaccinated for Hib (*p* = 0.0009).

**Table 1:** Characteristics of the study population by time point of urine collection

	Controls		Cases					
			Hospital Admission time point			Convalescent time point		
	%	*N*	%	*N*	*p*-value^a^	%	*N*	*p*-value^b^
Number of children		296		201			153	
Urinary arsenic concentration (μg/L) (Median (Interquartile Range))	14.0 (4.5–41.0)	296	24.0 (8.0–64.0)	201	<0.0001	16.0 (7.0–48.0)	153	0.26
Urinary creatinine concentration (mg/ dl) (Median (Interquartile Range))	22.4 (13.1–38.4)	296	48.1 (26.0–77.5)	201	<0.0001	19.1 (11.5–35.2)	153	0.14
Gender								
Female	54 %	161	57 %	115	0.01	64 %	98	0.01
Age (Months)								
Median (Interquartile Range) (Months)	10.0 (5.0–20.0)	296	13.0 (6.0–23.0)	201	0.88	12.0 (6.0–22.0)	153	0.90
0–5	33 %	99	25 %	50		24 %	36	
6–11	18 %	53	28 %	56		23 %	35	
12–23	27 %	80	25 %	50		32 %	49	
24–59	22 %	64	22 %	45		22 %	33	
Breastfed in the prior week								
Exclusive	24 %	71	23 %	45	0.33	22 %	33	0.50
Mixed	55 %	161	56 %	112		59 %	89	
None	21 %	61	22 %	43		20 %	30	
Case definition								
Severe pneumonia	–	–	89 %	179		88 %	135	
Very severe pneumonia	–	–	11 %	22		12 %	18	
WHO weight for height								
z-score less than −2 SDs	11 %	33	21 %	43	0.002	20 %	31	0.01
z-score greater or equal to −2 SDs	89 %	259	79 %	158		80 %	122	
Paternal education								
No formal education	12 %	35	18 %	36	0.07	18 %	28	0.03
1–5 years	21 %	61	20 %	41		22 %	34	
5–10 years	47 %	139	49 %	99		49 %	75	
Greater than 10 years	20 %	59	12 %	25		10 %	16	
Number of individuals living in household (Median (Interquartile Range))	5 (4–7)	296	5 (4–6)	201	0.05	5 (4–6)	153	0.10
Main source of drinking water^c^								
Piped water	1 %	4	2 %	5	0.76	3 %	5	0.69
Tube well	98 %	288	95 %	191		72 %	145	
River, stream, pond, lake	1 %	4	2 %	4		2 %	3	
Other	0 %	0	<1 %	1		0 %	0	
Floor of household								
Natural floor (sand/earth/dung)	79 %	233	86 %	173	0.04	86 %	131	0.08
Finished floor (wood/tiles/cement/carpet)	21 %	63	14 %	28		14 %	22	
Time-lapsed between hospital admission and urine collection in hours (Median (Interquartile Range))	0.75 (0.5–1.1)	290	3.2 (2.1–5.0)	193	<0.0001	–	–	–

^a^Control and case at admission time point compared using a two sample t-test for continuous variables and chi-square test for categorical variables

^b^Control and case at convalescent time point compared using a two sample t-test for continuous variables and chi-square test for categorical variables

^c^Tub well compared to all other drinking water sources

Creatinine concentrations of the admission case urine samples were significantly higher than those of control (*p* = <0.0001) and case convalescent (*p* = <0.0001) urine samples. There was no significant difference in urinary creatinine concentrations between control and case convalescent urine samples (*p* = 0.14). Cases were present at the hospital facility for significantly longer than controls before a urine sample was provided (*p* = <0.0001) (median hours 3.2 vs. 0.75). Among the 153 cases, the spearman correlation coefficients between the urinary arsenic concentrations at the hospital admission and the convalescent time points was 0.73 (*p* <0.001). The p-value of the paired t- test of the urinary arsenic concentrations of the urine samples collected at the hospital admission and convalescent time points was 0.001.

The odds ratios (OR) for severe and very severe pneumonia in increasing quartiles of urinary arsenic concentrations at the hospital admission time point were 1.00 (reference), 1.75 (95 % confidence interval (CI): (0.90, 3.40)), 2.11 (95 % CI: (1.01, 4.34)), and 2.04 (95 % CI: (0.92, 4.51)), after adjustment for urinary creatinine concentrations, weight for height, breastfeeding, paternal education, age, and number of people in the household (Table 2). Furthermore, there was a significant association between a urinary arsenic concentration higher than the first quartile (6 μg/L or greater) at the hospital admission time point and increased odds of pneumonia in the crude (2.28 (95 % CI: 1.37, 3.79)) and adjusted models (1.88 (95 % CI: 1.01, 3.50)). For the sensitivity analysis at the hospital admission time point, the odds ratios for *severe* pneumonia and urinary arsenic concentrations adjusted for creatinine were 1.00 (reference), 2.12 (95 % confidence interval (CI): (1.04, 4.32)), 2.35 (95 % CI: (1.07, 5.17)), and 2.36 (95 % CI: (1.02, 5.42)), after adjustment for study covariates.

**Table 2:** Crude and adjusted odds ratios of case and control urine samples at the hospital admission timepoint* (urinary creatinine in adjusted model)

Hospital Admission Time point								
All pneumonia cases (severe and very severe)								
Total urinary arsenic (μg/L)	Case urine samples (*N* = 153)		Control urine samples (*N* = 296)		Crude OR	95 % CI	Adjusted OR	95 % CI
	No.	%	No.	%				
0–5.9	23	15 %	85	29 %	1.00	Reference	1.00	Reference
6.0–16.9	37	24 %	77	26 %	1.78	0.97, 3.25	1.75	0.90, 3.40
17.0–50.9	43	28 %	69	23 %	2.30	1.27, 4.19	2.11	1.01, 4.34
Greater or equal to 51.0	50	33 %	65	22 %	2.84	1.58, 5.13	2.04	0.92, 4.51
Only severe pneumonia cases (sensitivity analysis)								
Total urinary arsenic (μg/L)	Case urine samples (*N* = 153)		Control urine samples (*N* = 296)		Crude OR	95 % CI	Adjusted OR	95 % CI
	No.	%	No.	%				
0–5.9	17	13 %	85	29 %	1.00	Reference	1.00	Reference
6.0–16.9	35	26 %	77	26 %	2.73	1.18, 4.38	2.12	1.04, 4.32
17.0–50.9	38	28 %	69	23 %	2.75	1.43, 5.30	2.35	1.07, 5.17
Greater or equal to 51.0	45	33 %	65	22 %	3.46	1.82, 6.60	2.36	1.02, 5.42

*Odds ratio were adjusted for weight for height (z-score less than −2 SDs), breastfeeding in the prior week (exclusive, mixed, none), paternal education, age, number of people living in the household (log transformed), and *urinary creatinine*

For the convalescent time point, the adjusted ORs for pneumonia in increasing quartiles of urinary arsenic were 1.00 (reference), 2.25 (95 % CI: 1.23, 4.11), 2.29 (95 % CI: 1.17, 4.47), and 2.56 (95 % CI: 1.27, 5.15) (Table 3). In addition, there was a significant association between a urinary arsenic concentration higher than the first quartile (6 μg/L or greater) at the convalescent time point and increased odds of pneumonia in the crude (1.65 (95 % CL: 1.03, 2.65)) and adjusted models (2.32 (95 % CI: 1.33, 4.02).

**Table 3:** Crude and adjusted odds ratios of case and control urine samples at the convalescent time point* (urinary creatinine in adjusted model)

Convalescent Time point								
All pneumonia cases (severe and very severe)								
Total urinary arsenic (μg/L)	Case convalescent urine samples (*N* = 153)		Control urine samples (*N* = 296)		Crude OR	95 % CI	Adjusted OR	95 % CI
	No.	%	No.	%				
0–5.9	30	20 %	85	29 %	1.00	Reference	1.00	Reference
6.0–14.9	44	29 %	67	23 %	1.86	1.06, 3.27	2.25	1.23, 4.11
15.0–41.9	39	25 %	72	24 %	1.53	0.87, 2,71	2.29	1.17, 4.47
Greater or equal to 42.0	40	26 %	72	24 %	1.57	0.89, 2.78	2.56	1.27, 5.15
Only severe pneumonia cases (sensitivity analysis)								
Total urinary arsenic (μg/L)	Case convalescent urine Samples (*N* = 153)		Control urine samples (*N* = 296)		Crude OR	95 % CI	Adjusted OR	95 % CI
	No.	%	No.	%				
0–5.9	24	18 %	85	29 %	1.00	Reference	1.00	Reference
6.0–14.9	39	29 %	67	23 %	2.06	1.13, 3.76	2.56	1.35, 4.85
15.0–41.9	38	28 %	72	24 %	1.87	1.03, 3.41	2.80	1.39, 5.63
Greater or equal to 42.0	34	25 %	72	24 %	1.67	0.91, 3.08	2.89	1.37, 6.08

*Odds ratio were adjusted for weight for height (z-score less than −2 SDs), breastfeeding in the prior week (exclusive, mixed, none), paternal education, age, number of people living in the household (log transformed), and *urinary creatinine*

The ORs for total urinary arsenic *unadjusted* for urinary creatinine are shown in Tables 4 and 5. At the hospital time point the adjusted ORs for pneumonia in increasing quartiles of urinary arsenic were 1.00 (reference), 2.38 (95 % CI: (1.25, 4.53)), 3.77(95 % CI: (1.88, 7.56)), and 4.42 (95 % CI: (2.30, 9.67)) (Table 4). For convalescent time point, the adjusted ORs for pneumonia in increasing quartiles of urinary arsenic were 1.00 (reference), 2.10 (95 % confidence interval (CI): (1.16, 3.81)), 1.97 (95 % CI: (1.03, 3.79)), and 2.03 (95 % CI: (1.04, 3.97)) (Table 5). There was also a significant association between a urinary arsenic concentration higher than the first quartile (6 μg/L or greater) at the hospital admission (OR: 3.12 (95 % CI: 1.74, 5.61)) and convalescent time point (OR: 2.05 (95 % CI: 1.20, 3.51) in the adjusted models.

**Table 4:** Crude and adjusted odds ratios of case and control urine samples at the hospital admission timepoint (without urinary creatinine adjustment)*

Hospital Admission Time point								
All pneumonia cases (severe and very severe)								
Total urinary arsenic (μg/L)	Case urine samples (*N* = 153)		Control urine samples (*N* = 296)		Crude OR	95 % CI	Adjusted OR	95 % CI
	No.	%	No.	%				
0–5.9	23	15 %	85	29 %	1.00	Reference	1.00	Reference
6.0–16.9	37	24 %	77	26 %	1.78	0.97, 3.25	2.38	1.25, 4.53
17.0–50.9	43	28 %	69	23 %	2.30	1.27, 4.19	3.77	1.88, 7.56
Greater or equal to 51.0	50	33 %	65	22 %	2.84	1.58, 5.13	4.72	2.30, 9.67
Only severe pneumonia cases (sensitivity analysis)								
Total urinary arsenic (μg/L)	Case urine samples (*N* = 153)		Control urine samples (*N* = 296)		Crude OR	95 % CI	Adjusted OR	95 % CI
	No.	%	No.	%				
0–5.9	17	13 %	85	29 %	1.00	Reference	1.00	Reference
6.0–16.9	35	26 %	77	26 %	2.27	1.18, 4.38	2.87	1.44, 5.72
17.0–50.9	38	28 %	69	23 %	2.75	1.43, 5.30	4.10	1.95, 8.63
Greater or equal to 51.0	45	33 %	65	22 %	3.46	1.82, 6.60	5.28	2.47, 11.26

*Odds ratio were adjusted for weight for height (z-score less than −2 SDs and z-score greater or equal to −2 SDs), breastfeeding in the prior week (exclusive, mixed, none), paternal education, age, and number of people living in the household (log transformed)

**Table 5:** Crude and adjusted odds ratios of case and control urine samples at the convalescent timepoint (without urinary creatinine adjustment)*

Convalescent Time point								
All pneumonia cases (severe and very severe)								
Total urinary arsenic (μg/L)	Case convalescent urine samples (*N* = 153)		Control urine samples (*N* = 296)		Crude OR	95 % CI	Adjusted OR	95 % CI
	No.	%	No.	%				
0–5.9	30	20 %	85	29 %	1.00	Reference	1.00	Reference
6.0–14.9	44	29 %	67	23 %	1.86	1.06, 3.27	2.10	1.16, 3.81
15.0–41.9	39	25 %	72	24 %	1.53	0.87, 2.71	1.97	1.03, 3.79
Greater or equal to 42.0	40	26 %	72	24 %	1.57	0.89, 2.78	2.03	1.04, 3.97
Only severe pneumonia cases (sensitivity analysis)								
Total urinary arsenic (μg/L)	Case convalescent urine samples (*N* = 135)		Control urine samples (*N* = 296)		Crude OR	95 % CI	Adjusted OR	95 % CI
	No.	%	No.	%				
0–5.9	24	18 %	85	29 %	1.00	Reference	1.00	Reference
6.0–14.9	39	29 %	67	23 %	2.06	1.13, 3.76	2.30	1.22, 4.32
15.0–41.9	38	28 %	72	24 %	1.87	1.03, 3.41	2.26	1.15, 4.46
Greater or equal to 42.0	34	25 %	72	24 %	1.67	0.91, 3.08	2.09	1.03, 4.23

*Odds ratio were adjusted for weight for height (z-score less than −2 SDs and z-score greater or equal to −2 SDs), breastfeeding in the prior week (exclusive, mixed, none), paternal education, age, and number of people living in the household (log transformed)

## Discussion

To our knowledge, this is the first study to assess the association between childhood arsenic exposure and pneumonia. We found a nearly two times higher odds of pneumonia for children with creatinine adjusted urinary arsenic concentrations higher than the first quartile (≥6 μg/L), when the hospital admission time point case and control urines were compared. An association between arsenic and respiratory infections in children at the low observed urinary arsenic concentrations, to our knowledge, has not been previously reported, and suggests that even low to moderate arsenic exposure can result in an increased risk of pneumonia in pediatric populations. Pneumonia is the leading cause of death globally for children under 5 years of age, causing an estimated 1.4 million deaths per year in this age group [18]. Therefore identifying risk factors for pneumonia provides an opportunity for interventions to reduce this disease burden in susceptible populations. These study findings provide preliminary evidence to support the hypothesis that arsenic exposure may be a potential risk factor for pneumonia in children under 5 years of age.

The design of this study overcomes many of the limitations of previous studies trying to assess the relationship between arsenic and respiratory health. Most previous studies have been ecologic, use self-reported or caregiver reported illness, and involve participants with comorbidities [14, 17, 19–21]. In the present study, the WHO classification of severe and very severe pneumonia was used by study physicians for diagnosis, providing a standardized case definition of illness. In addition, through the use of urinary arsenic as a biomarker we were able to measure both water and dietary contributions to pediatric arsenic exposure. This is in contrast to previous evaluations which rely on a household’s primary drinking water source or the mother’s urinary arsenic concentration as a proxy measure of childhood arsenic exposure [17, 20, 22, 48]. Furthermore, the nearly 2 year duration of the study allowed us to account for seasonal variations in pneumonia incidence, and the inclusion of the convalescent time point provided further validation of our findings at the hospital admission time point.

In our sensitivity analysis removing very severe pneumonia cases from the regression models increased the strength of the association between arsenic and pneumonia in all models. A significant association was found between urinary arsenic and severe pneumonia at both the hospital admission *and* convalescent time points for all quartiles compared. These findings suggest that there may be a stronger association between severe pneumonia and urinary arsenic than for very severe pneumonia. Future studies should use a larger sample size to investigate the association between arsenic exposure and very severe pneumonia in pediatric populations.

There was only a significant OR found between the first and the third quartile when creatinine-adjusted urinary arsenic concentrations and pneumonia were compared at the hospital admission time point, in contrast to significance for all quartiles at the convalescent time point. We suspect this is due to case children drinking less water than normal during their illness or water losses due to fever, elevated respiratory rate, and vomiting associated with their illness. Consistent with this explanation, there were significantly elevated urinary creatinine concentrations, a marker of dehydration, in case children during the hospital admission compared to the convalescent time point [49]. In addition, cases took significantly longer than controls to produce a hospital admission time point urine sample. We suspect the high ORs found in Table 4 when urinary arsenic concentrations at the hospital time point unadjusted for creatinine are compared is also attributed to case children being dehydrated, and therefore having more concentrated urine samples with higher arsenic concentrations. This large effect size was substantially reduced in Table 2 when an adjustment for creatinine was used, a measure of hydration status. Therefore the dehydration status of study children is a potential confounder of the association between arsenic and pneumonia at the hospital admission time point, and the convalescent time point urine sample is likely a better reflection of the case child’s urinary arsenic concentration after they recovered from their illness.

Beyond respiratory infections, there is also a growing body of literature demonstrating that early life arsenic exposure is associated with a wide variety of adverse health outcomes later in life. A recent cross-sectional study in Chile reported that early life arsenic exposure was associated with reduced forced expiratory volume measured in one second and lower forced vital capacity nearly 40 years after the high exposure ended [4]. Consistent with this, ecological studies in Chile have found significant associations between early life arsenic exposure and increased risk of lung cancer [50], kidney cancer mortality [51], and childhood liver cancer mortality [52]. Furthermore, there is a growing body of literature demonstrating an association between early life arsenic exposure and decreased cognitive and motor function in children [53–62]. These studies emphasize the need for effective arsenic mitigation strategies to reduce early life arsenic exposures. There have been several attempts to lower arsenic exposure in Matlab, Bangladesh through screening of arsenic in wells, the distribution of pond sand filters, and the installation of arsenic removal plants [63–65]. However, many individuals in Matlab remain arsenic exposed [64, 66]. Previous intervention studies have found that screening of wells for arsenic when combined with arsenic education can lower arsenic exposure, as evidenced by significant reductions in urinary arsenic concentrations [67, 68]. Therefore this could be a potential intervention approach to implement in Matlab, to reduce arsenic exposure in susceptible pediatric populations.

This study has several limitations. First, we lacked prospective data on the arsenic exposure history of study children and information on the child’s residential history. Instead, we relied on urinary arsenic at selected time points as a proxy of their lifetime exposure history. Therefore we may have misclassified the exposure histories of some study children. However, potential misclassification of exposure is unlikely to be differential by disease status in the case convalescent and control urine samples. The literature suggests that spot urine is a good proxy measurement of arsenic exposure *if* the source of drinking water remains constant [40–43]. Future studies should be conducted using prospective cohort studies in arsenic affected areas to investigate the causality of our observed association between pneumonia and urinary arsenic at low concentrations. In addition these studies should collect a residential history for each study child to determine the amount of time they have spent using water sources and the arsenic concentrations of these water sources should be measured. Second, we focused on severe and very severe pneumonia cases and therefore cannot draw conclusions on the association between arsenic and less severe pneumonia. Third, this is a facility based study and therefore the cases included may not be representative of pneumonia cases that do not report to the health facility, which comprise a large fraction of the pneumonia cases globally [69]. To assess the association between arsenic and pneumonia more fully would optimally require community-based studies. Finally, we do not have arsenic data from the primary drinking water source used by study children therefore we cannot associate water arsenic concentrations with pneumonia events.

## Conclusions

We observed a nearly two times higher odds of pneumonia for children with creatinine adjusted urinary arsenic concentrations greater than the first quartile (≥6 μg/L). This novel finding suggests that low to moderate arsenic exposure may be a risk factor for pneumonia in children under 5 years of age. Many other countries around the world are also affected by elevated levels of arsenic in groundwater sources such as India, Nepal, Vietnam, Cambodia, Mongolia, Taiwan, China, Chile, Argentina, Peru, Bolivia, Mexico, and the United States. Therefore the findings from this study have implications that are relevant globally and would benefit from future confirmatory evaluations.

## Additional file

Additional file 1: Table S1. Case characteristics by presence of convalescent urine sample. (DOC 91 kb)
