# Supplementary material for: Arsenic exposure is associated with pediatric pneumonia in rural Bangladesh: a case control study
Source: Environ Health. 2015 Oct 23;14:83. doi: 10.1186/s12940-015-0069-9 (PMC4619558; doi:10.1186/s12940-015-0069-9)
Supplement: Additional file 1: Table S1. — Case characteristics by presence of convalescent urine sample. (DOC 91 kb) [file 12940_2015_69_MOESM1_ESM.doc]

**Additional file 1: Table S1.** Case Characteristics by Presence of Convalescent Urine Sample

|  | **Convalescent Urine Sample Available** | | **Convalescent Urine Sample Missing** | |  |
| --- | --- | --- | --- | --- | --- |
|  | **%** | **N** | **%** | **N** | **p-value^1^** |
| **Number of Children** |  | 153 |  | 48 |  |
| **Hospital Time point: Urinary Arsenic Concentration (µg/L)** (Median (Interquartile Range))^2^ | 26.0 (9.0-66.0) | 153 | 20.0 (7.0-60.5) | 48 | 0.36 |
| **Hospital Time point: Urinary Creatinine Concentration (mg/dl)** (Median (Interquartile Range))^2^ | 45.2 (26.0-73.9) | 153 | 56.7 (26.1-89.9) | 48 | 0.47 |
| **Female** | 64% | 98 | 63% | 30 | 0.85 |
| **Age (Months)** |  |  |  |  |  |
| Median (Interquartile Range) | 12.0 (6.0-22.0) | 153 | 13.5 (7.0-25.0) |  | 0.45 |
| 0-5 | 24% | 36 | 21% | 10 |  |
| 6-11 | 23% | 35 | 19% | 9 |  |
| 12-23 | 32% | 49 | 35% | 17 |  |
| 24-59 | 22% | 33 | 25% | 12 |  |
| **Breastfed in the prior week** |  |  |  |  |  |
| Exclusive | 22% | 33 | 21% | 8 | 0.78 |
| Mixed | 59% | 89 | 64% | 25 |  |
| None | 20% | 30 | 15% | 6 |  |
| **Case Definition** |  |  |  |  |  |
| Severe Pneumonia | 88% | 135 | 92% | 44 | 0.51 |
| Very Severe Pneumonia | 12% | 18 | 8% | 4 |  |
| **WHO Weight for Height** |  |  |  |  |  |
| z-score less than -2 SDs | 20% | 31 | 25% | 12 | 0.48 |
| z-score greater or equal to -2 SDs | 80% | 122 | 75% | 36 |  |
| **Paternal Education** |  |  |  |  |  |
| No Formal Education | 18% | 28 | 17% | 8 | 0.37 |
| 1-5 years | 22% | 34 | 15% | 7 |  |
| 5-10 years | 49% | 75 | 50% | 24 |  |
| Greater than 10 Years | 10% | 16 | 19% | 9 |  |
| **Number of Individuals Living in Household** (Median (Interquartile Range)) | 5 (4-6) | 153 | 5 (4-6) | 48 | 0.84 |
| **Main Source of Drinking Water^3^** |  |  |  |  |  |
| Piped water | 3% | 5 | 0% | 0 | 0.77 |
| Tubewell | 95% | 145 | 96% | 46 |  |
| River, stream, pond, lake | 2% | 3 | 2% | 1 |  |
| Other | 0% | 0 | 0% | 1 |  |
| **Floor of Household** |  |  |  |  |  |
| Natural floor (sand/earth/dung) | 86% | 131 | 88% | 42 | 0.74 |
| Finished floor (wood/tiles/cement/carpet) | 14% | 22 | 12% | 6 |  |

^1^Case with and without a convalescent time point urine collected were compared using a two sample t-test for continuous variables and chi-square test for categorical variables 2. Hospital time point urine samples compared 3. Tubwell compared to all other drinking water sources
